# Towards an Energy Consumption Index for Deep Learning Models: A Comparative Analysis of Architectures, GPUs, and Measurement Tools

**DOI:** 10.3390/s25030846

**Published:** 2025-01-30

**Authors:** Sergio Aquino-Brítez, Pablo García-Sánchez, Andrés Ortiz, Diego Aquino-Brítez

**Affiliations:** 1 Department of Computer Engineering, Automation and Robotics, CITIC-UGR, University of Granada, 18014 Granada, Spain; pablogarcia@ugr.es (P.G.-S.); diegoaquino@correo.ugr.es (D.A.-B.); 2 Department of Communications Engineering, University of Málaga, 29071 Málaga, Spain; aortiz@ic.uma.es

**Keywords:** green computing, energy efficiency, machine learning, deep learning, convolutional neural network

## Abstract

The growing global demand for computational resources, particularly in Artificial Intelligence (AI) applications, raises increasing concerns about energy consumption and its environmental impact. This study introduces a newly developed energy consumption index that evaluates the energy efficiency of Deep Learning (DL) models, providing a standardized and adaptable approach for various models. Convolutional neural networks, including both classical and modern architectures, serve as the primary case study to demonstrate the applicability of the index. Furthermore, the inclusion of the Swin Transformer, a state-of-the-art and modern non-convolutional model, highlights the adaptability of the framework to diverse architectural paradigms. This study analyzes the energy consumption during both training and inference of representative DL architectures, including AlexNet, ResNet18, VGG16, EfficientNet-B3, ConvNeXt-T, and Swin Transformer, trained on the Imagenette dataset using TITAN XP and GTX 1080 GPUs. Energy measurements are obtained using sensor-based tools, including OpenZmeter (v2) with integrated electrical sensors. Additionally, software-based tools such as CarbonTracker (v1.2.5) and CodeCarbon (v2.4.1) retrieve energy consumption data from computational component sensors. The results reveal significant differences in energy efficiency across architectures and GPUs, providing insights into the trade-offs between model performance and energy use. By offering a flexible framework for comparing energy efficiency across DL models, this study advances sustainability in AI systems, supporting accurate and standardized energy evaluations applicable to various computational settings.

## 1. Introduction

Since the mid-20th century, global electricity demand has significantly increased, mainly driven by the use of fossil fuels, which contribute to greenhouse gas emissions and intensify environmental challenges on a global scale [1]. In response to these challenges, the Paris Agreement, established in 2015, aims to reduce these emissions and promote the transition to cleaner energy sources [2].

Technological advancements, particularly in Information and Communication Technologies (ICTs), play a key role in improving energy efficiency within the electrical sector. These technologies enable scalable and reliable communication infrastructures that support the integration of renewable energy sources, optimize demand response, and enhance the security and reliability of smart grids [3]. However, these advancements in ICT also bring new challenges [4]; for instance, the growth of Machine Learning (ML) and real-time data processing has led to an increased demand for computational resources, which in turn contributes to higher global energy consumption and associated environmental impacts.

ML algorithms, widely adopted to solve complex problems, require substantial computational resources, resulting in high energy consumption. This is mainly due to the complexity of ML architectures and the size of the datasets used during training, which extends the time and energy needed to achieve optimal performance [5]. Specifically, this growing demand for computational power is particularly noticeable in data centers, which have experienced an increase in electricity consumption [6]. Data centers handle key processes such as the training and inference of Artificial Intelligence (AI) algorithms, and their continuous expansion increases the energy footprint, raising concerns about the sustainability and efficiency of these systems [7].

In this research, we conduct an analysis of data collected separately during the training and inference phases of DL models, including classical and modern Convolutional Neural Networks (CNNs) [8], as well as the Swin Transformer [9] (Swin-T), to evaluate both their energy consumption and performance. Our analysis considers energy consumption during the training phase, where model parameters are optimized using input data through an iterative process, and the inference phase, where trained models process input data to generate predictions [8]. Energy consumption is measured using both hardware (OpenZmeter [10] v2) and software (CarbonTracker [11] v1.2.5 and CodeCarbon [12] v2.4.1) meters, ensuring an accurate assessment of the energy efficiency of DL models, defined as the ratio between performance and energy consumption. Furthermore, we validate several software-based energy meters to verify their accuracy. Proper calibration of these tools is crucial to ensuring data precision and optimizing energy usage in AI-driven environments.

In the context of related research, several studies have addressed the challenge of improving energy efficiency in computational systems, focusing on optimizing resources across a wide range of applications, from personal devices to supercomputers [13]. The methods used have varied, including improvements to specific algorithms, changes in hardware architectures [14], and programming techniques to reduce energy consumption [15]. Areas such as AI [16], data processing, and cloud computing (CC) [6] have received considerable attention, underscoring the importance of energy efficiency in high-demand contexts.

Despite recent advances, research faces ongoing challenges, such as the increasing complexity of computational models and the sustained growth in energy consumption [17]. Accurate measurement of energy consumption is key to identifying optimization opportunities, which has driven the development of specialized tools [15]. As research progresses, there is a trend toward using ML algorithms to predict and manage energy consumption, highlighting the need for new methodologies in this field [18].

Accurate tools for measuring energy consumption are essential for assessing the demands of hardware components such as processors, graphics cards, and other fundamental elements [19]. Recent studies emphasize the need to improve the accuracy and reliability of these tools through calibration processes and comparative analysis [20]. Energy consumption measurement tools exist in both hardware- and software-based forms; hardware tools provide direct, computational component-level measurements [21], while software-based tools enable analysis across different system layers.

MLPerf is a benchmarking suite designed to evaluate the performance of ML systems in training and inference tasks [22,23]. It covers domains such as vision, language, commerce, and reinforcement learning, evaluating tasks like classification, object detection, recommendation systems, speech recognition, and translation [22]. It allows performance comparisons across environments such as data centers, mobile devices, edge systems, and resource-constrained platforms (TinyML) [23]. The evaluation framework focuses on metrics such as training time, latency, and throughput under controlled conditions [24]. While it includes the option to report energy consumption metrics, these are not part of its primary evaluations and remain complementary within the MLPerf framework [23].

In this context, studies using software and hardware measurements reveal that hardware components, especially in data-center environments, exhibit unique energy profiles. Components like the CPU, memory, and storage display consumption patterns that vary with activity levels, enabling reductions of up to 40% from theoretical peak consumption through targeted optimization [25]. On the other hand, choices in software design, such as programming language selection, also have a significant impact on energy consumption and CO_2_ emissions [26]. Studies classify programming languages by their energy efficiency, generally indicating that faster languages tend to consume less energy. For example, Python, as an interpreted language, often requires more energy and has longer execution times due to line-by-line interpretation. In contrast, compiled languages like C are typically more efficient, as they translate directly into machine instructions, optimizing both speed and energy use [27].

Consequently, programming languages play a key role in energy efficiency. Low-level languages allow for greater control over system resources, facilitating specific optimizations that reduce energy use. In contrast, high-level languages, due to their level of abstraction, can introduce computational overhead that increases energy demands [28,29]. Research has shown that language choice can significantly influence energy consumption, execution time, and memory usage, depending on the application and hardware used [28]. In certain cases, languages with slower execution times achieve greater energy efficiency through better memory management, reducing redundant operations and enabling the compiler to optimize resource use [28].

The aforementioned research highlights the fundamental role of software, specifically the choice of programming language, in the energy efficiency of computational systems. However, with the increasing use of ML models, these challenges have expanded, as large-scale models require substantial computational resources and, consequently, result in significant energy consumption. The development of more energy-efficient technologies is no longer limited to hardware or programming language choices but also extends to the optimization of ML algorithms and architectures [30], which now account for a considerable portion of energy usage in high-performance systems [31].

The increasing environmental impact associated with large-scale training and inference stages of ML models, particularly in Deep Learning (DL), highlights the need for precise energy efficiency strategies. Such training and inferences demand substantial energy, which in turn contributes significantly to carbon emissions. This scenario requires the development of rigorous methodologies designed to both assess and mitigate these impacts effectively [32]. Key factors influencing energy consumption, including server location, training duration, and hardware configuration, have been identified across multiple studies, leading to the creation of tools that allow precise estimation and reduction in carbon emissions linked to ML training [20]. A range of approaches have been proposed to quantify energy use within ML applications [33], with comprehensive literature reviews cataloging the latest methodologies and specialized tools designed to track energy metrics throughout the stages of model training and execution [34].

In this context, measuring CNN energy consumption has become crucial. CNNs are widely used for their effectiveness, but require substantial computational resources, particularly during training and inference, making their energy efficiency a key concern to reduce environmental impact [35]. Studies show that CNN energy efficiency can vary widely depending on specific DL frameworks and hardware configurations, such as PyTorch [36] and TensorFlow [37], which influence both performance and energy demands.

Optimization techniques for CNNs are essential to improve energy efficiency while preserving model performance. Techniques like energy-aware pruning and Neural Architecture Search (NAS) enable CNNs to reduce energy usage without compromising accuracy [38]. Similarly, hyperparameter optimization and evolutionary algorithms have yielded energy-efficient CNN models by reducing computational demands [38,39]. Frameworks such as NeuralPower [40] enable precise energy consumption predictions, facilitating informed deployment decisions across different hardware platforms to optimize power efficiency. Additional approaches in CNN design further contribute to sustainable ML practices. For instance, energy-efficient methods in CNN layers help reduce power requirements, providing an efficient approach to feature extraction, while memory-optimized systems like EDEN [41] significantly lower energy consumption during inference, which is particularly beneficial for edge and mobile platforms [18]. Recent developments include lightweight architectures like the SCIFE block, which enhance information flow while reducing computational costs [42], and specialized hardware architectures like Morph-GCNX, which implement dynamic partitioning and optimization to improve energy efficiency in Graph Convolutional Networks (GCNs) [43]. The evolution of optimizers, from stochastic gradient descent to adaptive methods like Adam and SAM, has further contributed to balancing convergence speed with generalization, reducing the energy demands of training DL models [44].

Despite advances in measuring and optimizing ML models’ energy consumption, a key challenge persists: the lack of standardized metrics for consistently evaluating energy efficiency and carbon emissions. This standardization gap hinders objective and comparative assessments of environmental impact, complicating study comparability, and limiting the calibration of energy measurement tools [45]. Various metrics have been proposed, including floating-point operations (FPOs) [30], electricity consumption in kWh, CO_2_eq emissions, and execution time. However, each presents limitations: FPO, while useful for assessing computational load, does not reliably indicate actual energy use due to hardware variability and concurrent CPU/GPU usage [30]; kWh consumption and CO_2_eq emissions, though essential for environmental impact, often fail to capture efficiency differences across hardware and architectures [20,46,47]. Developing standards is crucial to consistently assess AI’s environmental impact and enhance energy efficiency.

Finally, this research proposes an efficiency index that integrates DL model performance with energy consumption measured separately during the training and inference stages, offering a unified criterion to evaluate both accuracy and sustainability. In this work, the proposed index is applied to DL models, including CNNs, transformer-based models, and hybrid networks, as use cases in multiclass classification problems. While the focus is on these specific architectures, the index demonstrates adaptability to a broader range of DL models. By combining the *Kappa Index* with the energy consumption measured separately during these stages, the *Kappa–Energy Index* ensures a precise evaluation of model performance, helping to avoid overestimations, especially in computationally intensive tasks. A higher *Kappa–Energy Index* indicates more efficiency, guiding architecture selection by balancing model accuracy and energy usage.

The main contributions of this paper are as follows:

1. Proposal of an energy consumption index to standardize the evaluation of energy efficiency in DL models during training and inference stages, offering a metric for objective comparisons across architectures.
2. Development of a combined methodology that uses both hardware and software energy meters to accurately measure energy consumption during the training and inference stages of DL models, enabling a precise evaluation of the energy-performance trade-off.
3. Analysis of the impact of heterogeneous hardware on the energy consumption and performance of DL models, examining how differences in hardware architectures affect the energy efficiency during both the training and inference stages of these models.
4. Validation and calibration of software energy meters to ensure accuracy in the assessment of the energy consumption during training and inference of DL models, aiming to achieve precise energy consumption measurements through software meters.

Section 2 describes the dataset, algorithms, and methodologies. Section 3 presents the experimental results. Section 4 discusses the results and findings. Section 5 concludes the study and outlines future research directions.

## 2. Materials and Methods

This section describes the dataset used in this research. We detail the dataset characteristics, the DL architectures used for multiclass classification problems, the hardware infrastructure, the tools and libraries employed, and the metrics applied to evaluate the performance of the DL models in the training and inference stages.

### 2.1. Data Description

This research used the Imagenette dataset, a 10-class subset of Imagenet [48] with 13,394 images, organized into 9469 training and 3925 testing images, stored in separate folders. Imagenette is a competitive dataset [49], enabling faster experimentation by reducing the time needed for the full Imagenet. Table 1 shows more details about this dataset.

**Figure 1:**
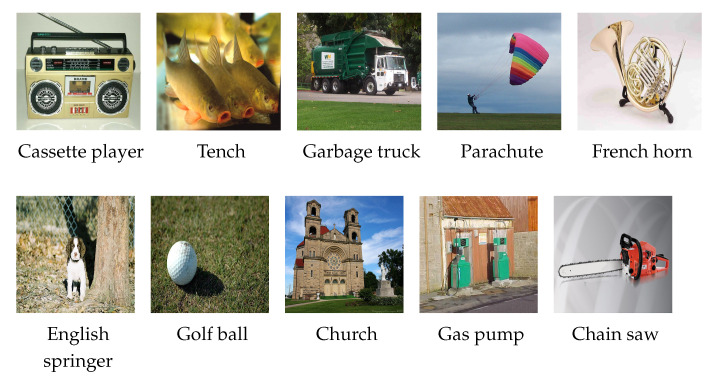
Images from the Imagenette dataset, a subset of ImageNet, showcasing 10 categories.

Figure 1 displays sample images from the Imagenette dataset, a subset of ImageNet, designed for efficient DL model testing in image classification tasks. It includes 10 different categories, allowing researchers to accelerate experimentation while preserving the complexity required for model evaluation.

### 2.2. Convolutional Neural Networks

CNNs are a subcategory of DL networks specifically engineered to process multidimensional structured data arrays, including spatial and temporal data. These models are particularly effective for tasks such as image recognition, object detection, and time series forecasting, where the data exhibit spatial hierarchies or temporal dependencies [16]. CNN architectures are multilayered, allowing for hierarchical feature learning. The foundational layer of CNNs is the convolutional layer [8], where the convolution operation, denoted as (f∗g)(x), between two functions f(x) and g(x), produces a third function s(x). Here, f(x) corresponds to the input, g(x) to the filter, and s(x) to the feature maps obtained by convolving f(x) and g(x), as defined in Equation (1).

(1) $$s\left(x\right)=\left(f*g\right)\left(x\right)=\sum_{i=1}^{n}f\left(i\right)\;\cdot \;g\left(x-i\right)$$

where *x* is a discrete variable representing arrays of numbers, and *n* corresponds to the filter size.

### 2.3. Transformer-Based Neural Networks

Transformers are a subcategory of DL networks designed to process sequential and structured data using the *self-attention* mechanism, which captures relationships between elements in a sequence independently of their position [50]. These multilayer architectures combine attention and *feedforward* layers to generate hierarchical representations [51]. The attention computation is based on the representations *Query* (*Q*), *Key* (*K*), and *Value* (*V*), as defined in Equation (2):

(2) $$Attention\left(Q,K,V\right)=\mathrm{Softmax}\left(\frac{QK^{T}}{\sqrt{d_{k}}}\right)V$$

where $d_{k}$ is the dimension of the keys. Transformers process all elements in parallel and use positional encoding to incorporate order information into the sequence, allowing them to capture both local and global relationships efficiently.

### 2.4. Implemented Deep Learning Architectures

This section outlines the DL architectures used in this study: AlexNet [52], VGG16 [53], ResNet18 [54], EfficientNet-B3 [55], Swin-T [9], and ConvNeXt-T [56]. These architectures are selected for their contributions to advancing DL and their effectiveness in image classification.

Convolutional Neural Networks, including both classical architectures such as AlexNet and VGG16, and modern architectures such as ResNet18 and EfficientNet-B3, serve as the primary case study. AlexNet set a benchmark by winning the 2012 ImageNet competition, demonstrating CNN capabilities, while VGG16, introduced in 2014, improved classification performance through a deeper architecture. ResNet, introduced in 2015, revolutionized DL with residual connections, enabling the training of much deeper networks and improving image recognition accuracy. EfficientNet-B3, introduced in 2019, employed a compound scaling method to balance depth, width, and resolution.

Additionally, the inclusion of Swin-T, introduced in 2021, brought transformer-based innovations to computer vision by combining hierarchical self-attention mechanisms with the ability to capture both local and global features efficiently. ConvNeXt-T, introduced in 2022, draws inspiration from CNN while incorporating elements from transformer-based models.

These six networks have been pivotal for computer vision. AlexNet pioneered GPU-based training and dropout regularization, with approximately 60 million parameters. VGG16 achieved high performance with its simple yet deep structure, consisting of about 138 million parameters. ResNet allowed for deeper architectures with innovative residual connections and has 11 million parameters. EfficientNet-B3 employed compound scaling to balance depth, width, and resolution, with approximately 12 million parameters. Swin-T leverages hierarchical transformers with around 28.2 million parameters, while ConvNeXt-T, with 28.6 million parameters, bridges the gap between CNNs and transformer-based innovations. These qualities make them suitable for assessing performance and energy consumption in DL applications, leading to their selection for this study. Table 2 summarizes the number of parameters [57] and creation year of the evaluated DL architectures.

The next sections detail these architectures.

#### 2.4.1. AlexNet

AlexNet, proposed by Alex Krizhevsky et al. in [52], was developed to address large-scale image classification tasks, solidifying its impact by securing first place in the ImageNet Large-Scale Visual Recognition Challenge (ILSVRC) in 2012, a significant milestone in the evolution of DL.

**Figure 2:**
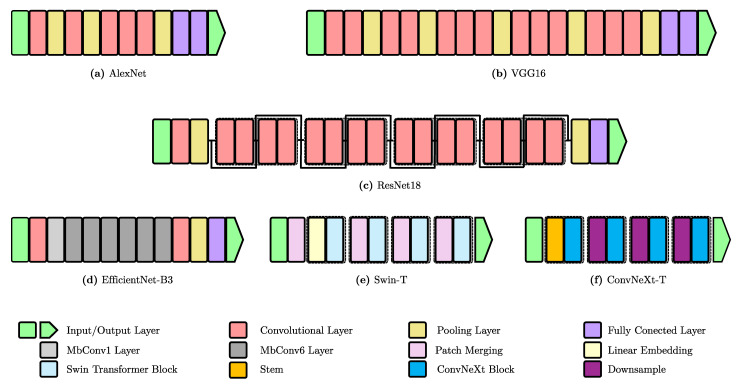
Comparison of different deep learning architectures: (**a**) AlexNet, (**b**) VGG16, (**c**) ResNet18, (**d**) EfficientNet-B3, (**e**) Swin-T, (**f**) ConvNeXt-T.

The architecture of AlexNet consists of five convolutional layers that employ convolutional filters to hierarchically extract features from the input images. Several of these layers are followed by pooling operations, a sampling technique that selects representative values within specific regions of the feature map. This process retains essential information while effectively reducing spatial dimensionality and computational demands. The final three layers include two fully connected layers followed by an output layer, which is also fully connected and responsible for performing the final classification. The architecture of AlexNet is shown in Figure 2a.

#### 2.4.2. VGG16

VGG16 [53] is a CNN architecture widely used in computer vision, particularly for image classification tasks. The architecture consists of 16 weight layers, organized in a sequence of convolutional, pooling, and fully connected layers. The main characteristic of VGG16 is its use of 3 × 3 convolutional filters with a stride of 1 and padding of 1 across all convolutional layers, enabling the model to capture spatial patterns at a consistent scale. This design choice reduces the number of parameters and improves the model’s generalization capability without compromising detailed feature extraction. Compared with earlier architectures with larger filters, VGG16 employs smaller filters at deeper layers, achieving higher model expressiveness.

Max-pooling layers with 2 × 2 filters and a stride of 2 pixels are applied after each convolutional block to reduce the spatial resolution of the feature maps. This pooling process not only helps manage data complexity but also introduces invariance to small spatial variations, a key factor in computer vision tasks. The fully connected layers at the end of the network consolidate the features extracted across previous layers, allowing for accurate classification at the output layer. The architecture of VGG16 is presented in Figure 2b.

#### 2.4.3. ResNet18

Residual Networks (ResNets) [54] introduce a critical architectural enhancement through residual connections, which effectively address the vanishing gradient problem inherent in Deep Neural Networks (DNNs). These residual connections, also referred to as skip connections, enable identity mappings that allow information to bypass one or more layers, ensuring that gradients can flow uninterrupted across multiple layers during backpropagation. This architecture allows ResNets to train networks of considerable depth without degradation, as gradients can propagate more effectively, thus mitigating the issues of gradient vanishing or explosion.

Each residual block in ResNet typically consists of two or three convolutional layers followed by batch normalization and ReLU activation, with the output added element-wise to the input via the residual connection. The ResNet family includes variants such as ResNet18, ResNet34, and ResNet50, where the number signifies the total depth in terms of convolutional layers, with deeper versions (e.g., ResNet152) leveraging additional residual blocks to achieve greater model capacity while maintaining manageable computational demand.

In this research, ResNet18 is selected, featuring 18 layers. The architecture incorporates skip connections that allow gradients to flow more easily during backpropagation, enhancing its performance in DL tasks. The architecture is shown in Figure 2c.

#### 2.4.4. EfficientNet-B3

EfficientNet-B3 [55] is a CNN architecture used for image classification tasks. The architecture combines convolutional layers, MBConv blocks, and fully connected layers. MBConv blocks employ inverted residual convolutions with skip connections, reducing dimensionality while optimizing resource usage. Additionally, the Swish activation function is utilized, which improves gradient propagation compared with traditional activation functions like ReLU.

The EfficientNet-B3 model applies compound scaling, which uniformly adjusts the depth, width, and resolution of the network to maximize performance across various resource configurations. Initial layers extract general features through standard convolutions, while MBConv blocks focus on specific patterns at greater depths. A global average pooling (GAP) layer reduces the number of parameters before the fully connected layers. The architecture of EfficientNet-B3 is presented in Figure 2d.

#### 2.4.5. Swin-T

The Swin Transformer [9] is a state-of-the-art architecture that leverages transformer-based innovations. Unlike traditional CNNs, Swin-T employs a hierarchical structure with Shifted Window Attention, which divides images into non-overlapping windows and computes self-attention within each window. This mechanism enables Swin-T to capture local and global dependencies while maintaining scalability to higher image resolutions.

The hierarchical nature of Swin-T allows the model to progressively aggregate information at different scales, similar to the pooling operations in CNNs. At each stage, feature maps are downsampled, enabling Swin-T to learn multilevel representations.

Each stage in Swin-T consists of Swin Transformer Blocks, where the combination of MultiHead Self-Attention (MHSA) and MultiLayer Perceptrons (MLPs) ensures robust feature extraction. The residual connections in these blocks further enhance gradient flow during backpropagation. The architecture of Swin-T is shown in Figure 2e.

#### 2.4.6. ConvNeXt-T

ConvNeXt-T [56] is a hybrid convolutional architecture inspired by both traditional CNNs and modern transformer-based models. It reimagines the standard convolutional block by integrating features such as depthwise separable convolutions, Layer Normalization, and inverted bottleneck designs.

The architecture of ConvNeXt-T replaces batch normalization with Layer Normalization, optimizing gradient flow and reducing memory overhead. Additionally, ConvNeXt-T introduces Large Kernel Convolutions to better capture long-range dependencies, bridging the gap between the local receptive fields of CNNs and the global feature extraction capabilities of transformers. These design choices improve its ability to generalize across diverse datasets.

The architecture comprises four stages, each consisting of ConvNeXt blocks. These blocks leverage a combination of depthwise and pointwise convolutions to reduce the number of parameters. The final stage consolidates features for classification through a GAP layer followed by fully connected layers. The architecture of ConvNeXt-T is shown in Figure 2f.

### 2.5. Performance Evaluation Metrics

This section details the metrics used to evaluate the performance of the predictive models and the units of measurement used to record energy consumption during model training.

#### 2.5.1. Model Performance Evaluation

The *Kappa Index* [58], or Cohen’s Kappa, is used in this work as it evaluates the agreement between the model’s predictions and the actual labels, applicable in both binary and multiclass classification problems. Unlike accuracy, *Kappa Index* adjusts its values by considering the agreement that could occur by chance, offering a more precise evaluation of the model’s performance. It is defined as

(3) $$k=\frac{p_{0}-p_{c}}{1-p_{c}}$$

where $p_{0}$ is the proportion of observed agreements and $p_{c}$ is the proportion of agreements expected by chance. The index ranges from −1 to 1, with 1 indicating perfect agreement, 0 representing agreement equivalent to chance, and negative values suggesting worse-than-chance performance.

#### 2.5.2. Measuring Energy Consumption

Energy consumption is measured in kilowatt-hours (kWh) [59], which represents the use of 1 kilowatt of power over 1 h. In joules (J), 1 kWh corresponds precisely to 3,600,000 J, calculated as 1 kilowatt (1000 watts) sustained over 3600 s. The kWh is a standard unit commonly used to quantify electrical energy consumption across various contexts [13,33]. It is defined as

(4) kWh=P×t

where *P* is the power in kilowatts (kW) and *t* is the time in hours (h).

#### 2.5.3. DL Model Performance over Energy Consumption

The *Kappa–Energy Index* (KEI), proposed in this research, specifies the relation between model performance and energy consumption during the training or inference of DL models. It is defined as

(5) $$\mathrm{Kappa}–\mathrm{EnergyIndex}=\frac{\mathrm{Kappa}\mathrm{Index}}{\mathrm{Energy}\mathrm{Consumption}}$$

where the *Kappa Index*, denoted as *k* in Equation (3), represents the model’s performance, and the Energy Consumption, denoted as kWh in Equation (4), represents the kilowatt-hours used during model training or inference. It should be noted that the KEI index is applicable to larger systems, including data center servers, future GPU architectures, and ML servers, provided that tools are available to measure energy consumption relevant to the object of study. Furthermore, the index can be extended to distributed environments by aggregating energy data from multiple nodes or GPUs, facilitating the evaluation of energy efficiency in complex computational systems.

The *Kappa Index* is selected over other metrics, such as accuracy, due to its robustness and interpretative advantages. Unlike simple accuracy, the *Kappa Index* accounts for chance agreement, enhancing reliability in performance assessment. It is especially useful for imbalanced classes, providing a conservative performance measure. Additionally, Table 3 presents the interpretation of the *Kappa Index* on a standardized scale, with established benchmarks that categorize levels of agreement [60].

It is important to indicate that a higher KEI index reflects greater energy efficiency. In summary, a higher index indicates DL models achieving a better trade-off between their performance and energy consumption.

#### 2.5.4. Measuring CO_2_ Emissions

CO_2_ emissions are measured in kilograms of CO_2_ equivalents (CO_2_eq), which represent the amount of carbon dioxide released based on the energy consumption of the computational infrastructure [61]. The calculation of CO_2_ emissions is performed by multiplying the carbon intensity of electricity (*C*, in grams of CO_2_ per kilowatt-hour) by the energy consumed (*E*, in kilowatt-hours). It is important to indicate that CodeCarbon ${\mathrm{CO}}_{2}$ emissions calculation is described in Section 2.6. In this way, providing an accurate calculation of the total CO_2_ emissions in kilograms of CO_2_ equivalents (CO_2_eq):

(6) $${\mathrm{CO}}_{2}\mathrm{eq}=C\times E$$

where *C* is the carbon intensity and *E* is the energy consumed in kilowatt-hours.

### 2.6. Energy Consumption Meters

This section outlines the sensor-based tools used to analyze the energy consumption of the algorithms, ensuring precise and reliable measurements. Energy data are collected using hardware and software tools: OpenZmeter (oZm) [10], a hardware-based device equipped with integrated electrical sensors to directly measure consumption, and software-based tools such as CodeCarbon [61] and Carbontracker [11], which retrieve energy consumption data from computational component sensors. OpenZmeter is chosen for its ability to measure energy consumption and its cost. It supports autonomous operation and cloud communication for energy monitoring [12]. On the other hand, the CodeCarbon library is chosen for its capability to measure energy consumption and calculate CO_2_ emissions accurately [32]. Finally, Carbontracker estimates CO_2_ emissions and energy consumption, with results aligning with wattmeter measurements across various infrastructures [32]. A more detailed description of each energy meter is provided in the following sections.

**Figure 3:**
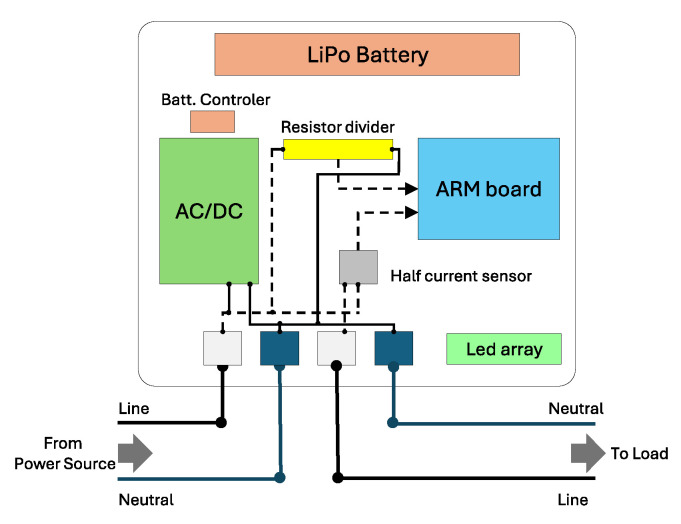
Schematic diagram of the OpenZmeter. It includes an ARM board, AC/DC converter, LiPo battery, and sensors to measure electrical parameters from the power source to the load. Image adapted from [10].

**Figure 4:**
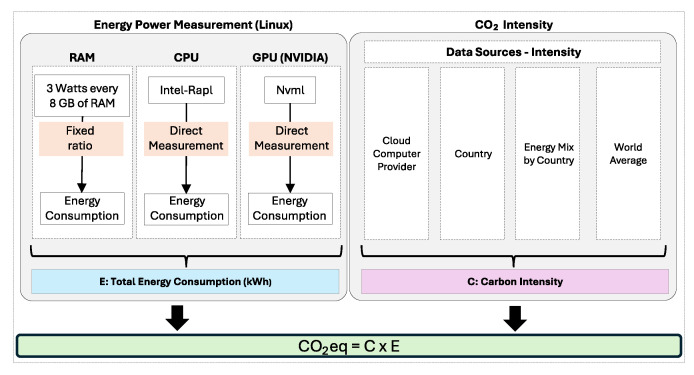
CodeCarbon [61] energy measurement process. Energy consumption (*E*, in kWh) is estimated using RAM (3 W/8 GB), CPU (Intel-RAPL), and GPU (Nvml). Carbon intensity (*C*, in kgCO_2_eq/kWh) integrates data from cloud providers, country energy mixes, and world averages to compute CO_2_eq.

**Figure 5:**
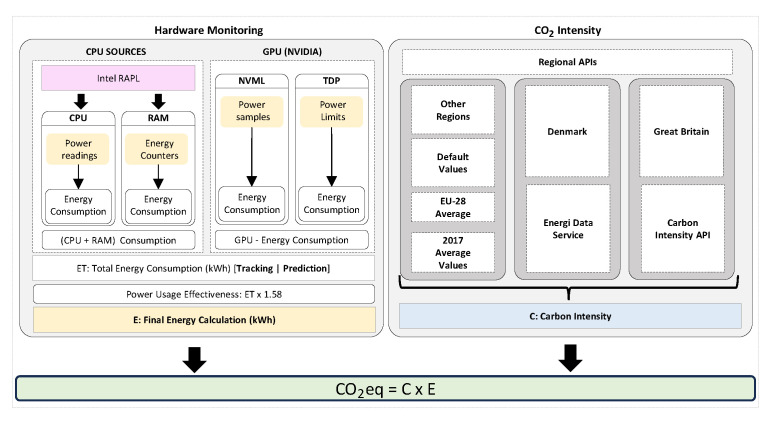
Carbontracker [11] process for estimating energy consumption (*E*, kWh) using CPU (Intel RAPL), GPU (Nvml, TDP), and RAM data, adjusted by PUE. Carbon intensity (*C*, kgCO_2_eq/kWh) is obtained from regional APIs. Final emissions (CO_2_eq) are calculated as $CO_{2}eq=C\times E$.

- OpenZmeter [10] is a low-cost, open-source, intelligent hardware energy meter and power quality analyzer. It measures reactive, active, and apparent energy, frequency, Root Mean Square (RMS) voltage, RMS current, power factor, phase angle, voltage events, harmonics up to the 50th order, and total harmonic distortion (THD). It records energy consumption in kilowatt-hours (kWh). The device includes a web interface and an API for integration. It can be installed in electrical distribution panels and features Ethernet, Wi-Fi, and 4G connectivity. Additionally, it offers remote monitoring and real-time alerts. Figure 3 shows the OpenZmeter diagram.
- CodeCarbon [61] is an open-source tool to measure and reduce software programs’ carbon footprint. It tracks energy consumption in kilowatt-hours (kWh) during code execution, accounting for hardware and data center location to calculate CO_2_ emissions. Carbon intensity can vary hourly and adapt to users’ location when real-time data from sources like the CO_2_ Signal API are accessible. For cloud computing, CodeCarbon uses Google Cloud Platform data (Mountain View, CA, USA), although Amazon (Seattle, WA, USA) and Microsoft (Redmond, WA, USA) do not provide specific carbon details for their data centers. For private infrastructures, CodeCarbon draws from “Our World in Data” when available or defaults to the energy mix of the user’s country from “globalpetrolprices.com” adjusting carbon intensity accordingly. When specific data are absent, a global average of 475 gCO_2_/kWh, based on the International Energy Agency (IEA), is applied. The tool also offers an Application Programming Interface (API) and Python libraries to integrate carbon monitoring into projects, along with reports and visualizations that consider data center locations. Energy consumption measurement focuses on key system components, specifically the GPU, RAM, and CPU. However, the tool does not account for other elements, such as storage, network, or peripherals, which leads to underestimations of total consumption. Figure 4 shows the CodeCarbon diagram.
- Carbontracker [11] is an open-source software tool for energy management in the training of DL models, allowing users to track and predict energy consumption and carbon emissions. It facilitates the adjustment of epochs to monitor consumption and can track the entire training process to provide accurate estimates. Noteworthy research utilizing Carbontracker includes a study cited in [62], which discusses how combining federated learning with transfer learning can enhance the classification of medical images in an energy-efficient and privacy-preserving manner. Another investigation [63] examines the relationship between the quality of generative audio models and their energy consumption. Figure 5 shows the Carbontracker diagram.

Table 4 provides a comparative overview of three tools used for tracking energy and carbon footprints in DL workflows: OpenZmeter, CodeCarbon, and Carbontracker. The tools differ in type, energy measurement, carbon tracking, and features. Each addresses specific aspects of monitoring energy use during model training and inference.

### 2.7. Computational Resources

The experiments being conducted in this research are performed using the cluster of the Biomedical Signal Processing, Computational Intelligence, and Communications Security (BIOSIP) research group at the University of Málaga, Spain. Exclusive access to the required computational resources is provided to ensure the successful execution of the experiments.

Table 5 details the architecture of the node used for the experiments. This setup includes the hardware components and software tools required to control the experimental environment, ensuring accurate energy measurements and reproducible results.

### 2.8. Experimental Setup

This section outlines the experiments that evaluate the energy consumption during the training of various DL architectures, as well as the inference stages of previously trained DL models. The architectures selected for evaluation include AlexNet, VGG16, ResNet18, EfficientNet-B3, Swin-T, and ConvNeXt-T, chosen for their relevance in the current research on DL Architectures. Energy consumption is measured using the OpenZmeter, a hardware energy meter used as reference, along with two software tools, Carbontracker and CodeCarbon, which record energy usage every 15 s, while the hardware meter collects data every second. Both software tools also provide CO_2_ emission estimates, either through predefined indices or by accessing renewable energy databases. In this study, the CO_2_ calculations are based on data specific to Andalucía, Spain, obtained solely through these software measurement tools.

In the experimental training setup, each DL architecture is trained up to 90 epochs, with a batch size of 64, and a learning rate of 0.001. These hyperparameters [8] are selected to perform comparable evaluations across different architectures, enabling an objective analysis of their energy demands. The corresponding values for each hyperparameter are detailed in Table 6.

The training is performed using two GPU models, the TITAN Xp and the GTX 1080, with each architecture being trained individually on a single GPU. The dataset used is Imagenette, split into 70% for training and 30% for testing. The training set is further divided into 80% for training and 20% for validation. The test set is used to evaluate the final performance of the models. Each experiment is repeated 15 times to ensure statistical validity, with an estimated training time limit of 20 min for each network, and a seed value of 1 is used to ensure consistent initial conditions across all runs.

**Figure 6:**
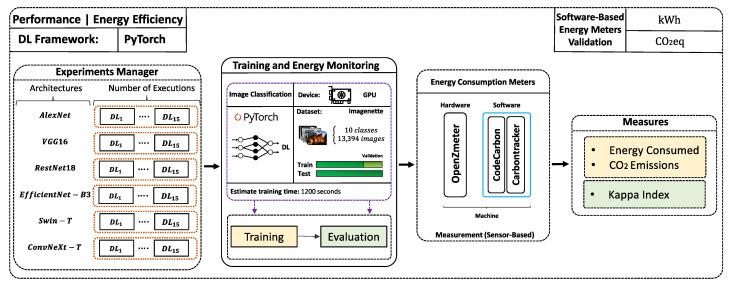
Experimental framework for evaluating the performance and energy consumption of AlexNet, VGG16, ResNet18, EfficientNet-B3, Swin-T, and ConvNeXt-T measured with OpenZmeter, CodeCarbon and Carbontracker.

Figure 6 shows the experimental setup designed to evaluate the performance and energy consumption of the evaluated DL architectures. Energy consumption and CO_2_ emissions are monitored using tools such as OpenZmeter, CodeCarbon, and Carbontracker, while the models are trained on the Imagenette dataset using GPUs.

Algorithm 1 presents the pseudo-code for evaluating model performance and energy consumption.    
**Algorithm 1:** Pseudo-code for Performance and Energy Evaluation
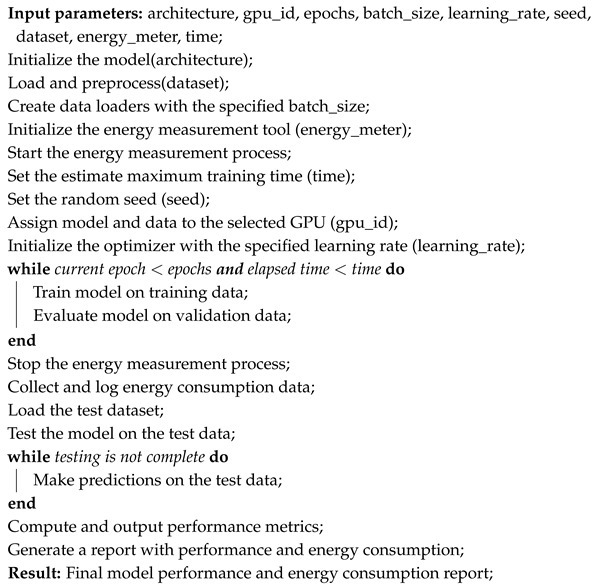


Finally, for the experimental inference setup, each previously generated DL model is evaluated on the same GPU used during its initial training. The dataset used consists of 10 classes, each containing 100 images. The complete inference process is repeated 15 times for each model, using the same dataset each time.

## 3. Results

In this section, we present the results obtained from the comparison of energy consumption measurement tools, including software and hardware energy meters, evaluating their precision and consistency in various hardware environments. The experiments are conducted using various GPUs, focusing on energy consumption during the training and inference phases of the selected DL models. Then, we evaluate the proposed *Kappa–Energy Index*, which relates the achieved *Kappa Index* of the DL models to their energy consumption in kWh. Finally, the results are subjected to statistical validation at each stage that requires it.

### 3.1. Energy Consumption and Meter Precision in DL Models Training Experiments

The experimental results for energy consumption and CO_2_ emissions during the training of various DL models on different GPUs are analyzed and presented. Energy meter tools are used to conduct a comparative analysis of energy consumption, execution times, and CO_2_ emission estimates, allowing for an assessment of the accuracy of software energy measurements compared with a hardware meter, which is used as a reference. Additionally, the section titled *Active Power Consumption During DL Models Training* provides detailed real-time active power consumption profiles for each architecture.

**Figure 7:**
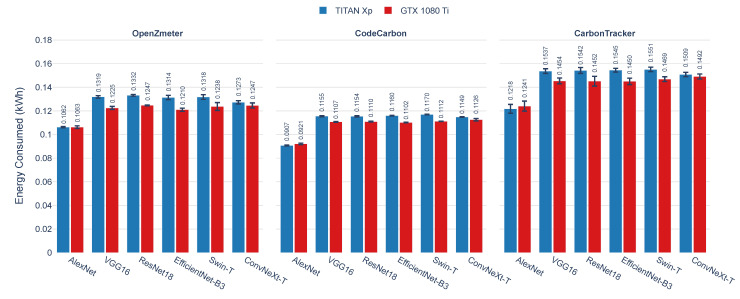
Energy consumption (kWh) during the training of AlexNet, VGG16, ResNet18, EfficientNet-B3, Swin-T, and ConvNeXt-T on GPUs TITAN Xp and GTX 1080 Ti. The graph compares the results from three energy measurement tools: OpenZmeter (energy meter reference), CodeCarbon, and Carbontracker.

Figure 7 presents the energy consumption in kilowatt-hours (kWh) for each DL model implemented and trained on both GPUs. Measurements from OpenZmeter, CodeCarbon, and Carbontracker are compared to highlight differences in their recorded values. OpenZmeter consistently provides energy consumption measurements across both GPUs used. While overall energy consumption is comparable among the DL models, it is noted that the TITAN Xp GPU consumes more energy than the GTX 1080 Ti, particularly for models such as VGG16, ResNet18, EfficientNet-B3, Swin-T, and ConvNeXt-T.

In comparison, CodeCarbon reports lower energy consumption values. However, despite these underestimations, its measurement trends are closely aligned with those of OpenZmeter, indicating similar consumption trends across GPUs. In contrast, Carbontracker records higher consumption values across all models, with notable discrepancies in the training of VGG16, ResNet18, EfficientNet-B3, Swin-T, and ConvNeXt-T, where it measures significantly higher energy use than OpenZmeter and CodeCarbon.

A consistent trend is observed where the GTX 1080 Ti exhibits lower energy consumption compared with the TITAN Xp in all DL models evaluated. This pattern is particularly evident in measurements from OpenZmeter and CodeCarbon, which show minimal variability in the energy consumption differences between GPUs. Carbontracker, while following a similar pattern, reports significantly higher consumption values overall, amplifying the observed gap between the TITAN Xp and GTX 1080 Ti.

**Figure 8:**
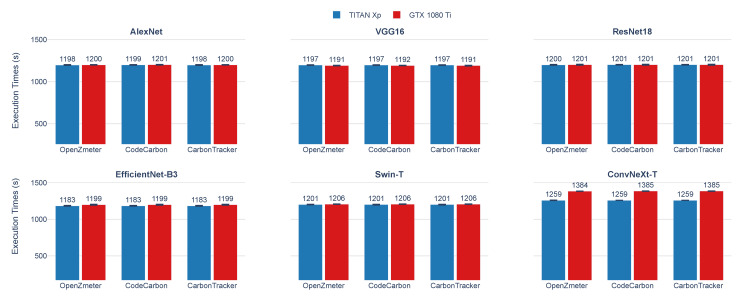
Average Execution times (s) during the training of AlexNet, VGG16, ResNet18, EfficientNet-B3, Swin-T, and ConvNeXt-T on GPUs TITAN Xp and GTX 1080 Ti. The graph compares the results from three energy measurement tools: OpenZmeter (energy meter reference), CodeCarbon, and Carbontracker.

In parallel, Figure 8 examines another aspect of energy consumption measurement: the execution times (s) during the training of the six DL models on the two GPUs. Small variations are observed in the training times recorded by each measurement tool (OpenZmeter, CodeCarbon, and Carbontracker). Although these discrepancies are limited to a few seconds and remain consistent, they are likely due to differences in the starting and ending points of the recording process used by each tool. The results indicate that execution times across both GPUs and measurement tools are consistent, with minimal differences observed for most architectures. For AlexNet and VGG16, times are nearly identical on the TITAN Xp and GTX 1080 Ti, reflecting similar processing capabilities. ResNet18 execution times are consistent across GPUs and tools. EfficientNet-B3 shows the lowest times on the GTX 1080 Ti, while on the TITAN Xp, times are comparable to other architectures. Swin-T demonstrates a slight increase in execution times on the GTX 1080 Ti compared with the TITAN Xp across all tools, with minimal variation. Conversely, ConvNeXt-T records the longest execution times among all models, with a more pronounced difference between the TITAN Xp and GTX 1080 Ti, particularly when measured by OpenZmeter.

Table 7 presents a detailed analysis of energy consumption and execution times for six DL models on two GPUs: TITAN Xp GPU and GTX 1080 Ti GPU. This analysis compares energy consumption results obtained through three measurement tools: OpenZmeter, CodeCarbon, and Carbontracker. Execution time and energy consumption values are presented as averages and standard deviations, allowing for an assessment of both central tendency and variability in performance.

The results indicate consistency in execution times across architectures for each GPU. However, differences in energy consumption estimates reveal the influence of the measurement tool on reported efficiency values. The comparison of OpenZmeter with CodeCarbon and Carbontracker enables the evaluation of the accuracy of software energy measurements. The low *p*-values demonstrate that the discrepancies among the tools are statistically significant, which confirms that each tool produces distinct energy consumption estimates under the same conditions.

Despite these differences, when observing the average energy consumption values for the architectures AlexNet, VGG16, ResNet18, EfficientNet-B3, Swin-T, and ConvNeXt-T, it is noted that CodeCarbon, although underestimating energy consumption compared with OpenZmeter, tends to be closer to the reference values. This suggests that CodeCarbon provides a more reliable estimate in terms of trends. On the other hand, Carbontracker demonstrates a systematic tendency to overestimate energy consumption across all architectures analyzed.

Overall, these results highlight the importance of validating software-based energy measurement tools against reference meters like OpenZmeter, particularly in scenarios where accuracy is critical. Additionally, architectural characteristics, as seen in ResNet18, EfficientNet-B3, Swin-T, and ConvNeXt-T, play a significant role in energy consumption, exceeding the values recorded for AlexNet and VGG16.

In this regard, CodeCarbon offers an additional feature for measuring CO_2_ emissions, which complements the energy consumption analysis. This feature provides a more comprehensive view of the environmental impact of DL models training by including greenhouse gas estimates. Quantifying CO_2_ emissions helps assess the energy cost in terms of electricity use and supports evaluations of sustainability and environmental impact.

**Figure 9:**
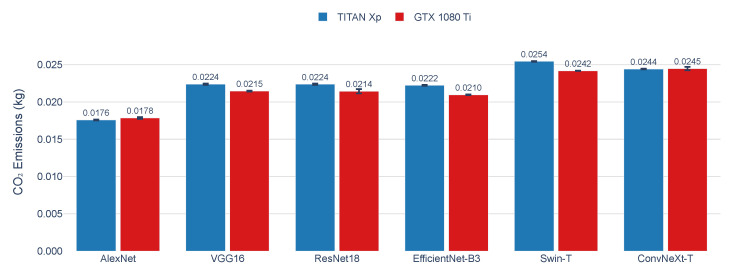
CO_2_ Emissions during the training of AlexNet, VGG16, ResNet18, EfficientNet-B3, Swin-T, and ConvNeXt-T on GPUs TITAN Xp and GTX 1080 Ti; results obtained using CodeCarbon.

Therefore, Figure 9 presents CO_2_ emissions (in kilograms) for the six DL models during experimental runs conducted on the TITAN Xp and GTX 1080 Ti GPUs. For AlexNet, emissions are the lowest among the architectures, with both GPUs producing nearly identical emissions, indicating no difference in CO_2_ emissions between the TITAN Xp and GTX 1080 Ti for this architecture. In VGG16, CO_2_ emissions increase compared with AlexNet, with the TITAN Xp producing approximately 5% more CO_2_ than the GTX 1080 Ti. Although this difference is minor, the emissions for both GPUs remain close for this architecture. A similar pattern is observed in ResNet18, with the TITAN Xp again emitting around 5% more CO_2_ than the GTX 1080 Ti. In EfficientNet-B3, the CO_2_ emissions on the TITAN Xp are approximately 5% higher than on the GTX 1080 Ti, slightly exceeding the approximately 5% observed for ResNet18. This corresponds to a 21% greater relative difference for EfficientNet-B3 compared with ResNet18. In Swin-T, CO_2_ emissions increase further, with the TITAN Xp emitting approximately 5% more CO_2_ than the GTX 1080 Ti. However, the overall emissions for both GPUs remain close, reflecting a consistent trend across architectures. Finally, ConvNeXt-T shows the highest emissions among all architectures, with the GTX 1080 Ti producing slightly higher CO_2_ emissions compared with the TITAN Xp. The difference, while minor, highlights the greater computational demand of this model. Overall, emissions from the two GPUs are almost equivalent, with the GTX 1080 Ti producing less CO_2_ in the more complex architectures.

#### Active Power Consumption During DL Models Training

**Figure 10:**
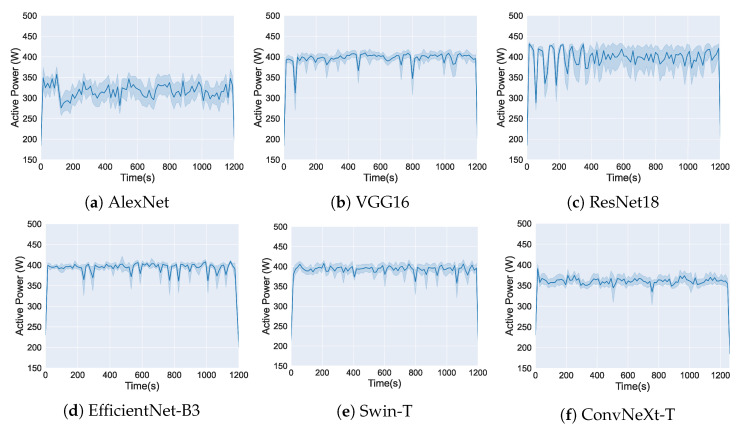
Active power consumption during the training of DL models on TITAN Xp using OpenZmeter. The solid lines show the average active power consumption over time, while the shaded areas indicate the variability of the data at each time point.

**Figure 11:**
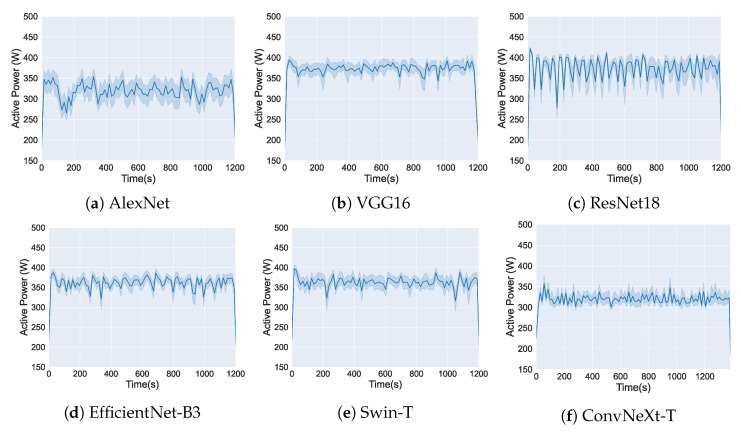
Active power consumption during the training of DL Models on GTX 1080 Ti using OpenZmeter. The solid lines show the average active power consumption over time, while the shaded areas indicate the variability of the data at each time point.

In this section, we analyze the active power consumption during the training of the six evaluated DL Models. Figure 10 and Figure 11 show the measurements obtained using the OpenZmeter hardware meter, showing the active power (W) over time (s) for the two used GPUs.

Figure 10 presents the results of power consumption during training on the TITAN Xp GPU. The six graphs correspond to each DL Models: (a) AlexNet, (b) VGG16, (c) ResNet18, (d) EfficientNet-B3, (e) Swin-T, and (f) ConvNeXt-T.

Figure 11 presents the active power consumption of the same DL models trained on a GTX 1080 Ti GPU. As in the previous figure, the graphs are organized as (a) AlexNet, (b) VGG16, (c) ResNet18, (d) EfficientNet-B3, (e) Swin-T, and (f) ConvNeXt-T.

As a result, Figure 10 and Figure 11 show that despite the different GPUs used, each DL model demonstrates a distinct energy consumption profile during the training process. While the choice of GPU affects the absolute consumption values, the patterns of energy utilization over time are influenced by the structure and design of each DL model.

### 3.2. Evaluation of Kappa–Energy Index for DL Models Training and Inference

In this section, the *Kappa–Energy Index* is used to evaluate the training and inference of DL Models on different GPUs.

#### 3.2.1. Evaluation of Kappa–Energy Index for DL Models Training

**Figure 12:**
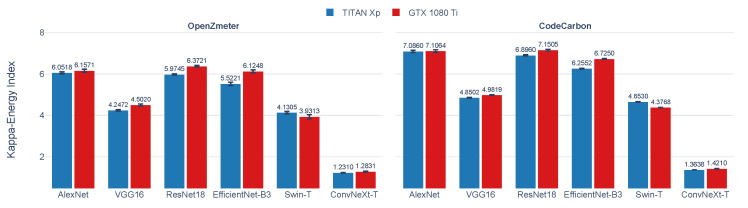
*Kappa–Energy Index* for AlexNet, VGG16, ResNet18, EfficientNet-B3, Swin-T, and ConvNeXt-T on TITAN Xp and GTX 1080 Ti GPUs, comparing OpenZmeter (hardware reference meter) and CodeCarbon (software meter) results.

In the context of DL models training analysis, Figure 12 presents the *Kappa–Energy Index* for DL models on two GPUs, comparing OpenZmeter with CodeCarbon. The *Kappa–Energy Index* represents the energy efficiency of each model-GPU combination, where higher values indicate greater efficiency. This comparison reveals variations in *Kappa Index* values between the hardware and software energy meters across the models and GPUs.

The GTX 1080 Ti consistently achieves higher *Kappa–Energy Index* values than the TITAN Xp across all evaluated architectures, indicating better energy efficiency for this GPU. Among the architectures, VGG16 exhibits the lowest index values compared with ResNet18 and EfficientNet-B3, reflecting its higher energy demands and architectural complexity. ResNet18 achieves the highest index values across all measurements, particularly on the GTX 1080 Ti, while EfficientNet-B3 shows comparable values, highlighting its energy-efficient design. Swin-T exhibits intermediate *Kappa–Energy Index* values, with the GTX 1080 Ti consistently outperforming the TITAN Xp. In contrast, ConvNeXt-T shows the lowest index values among all architectures, regardless of the GPU, emphasizing its higher energy demands due to its complexity.

Table 8 compares the *Kappa–Energy Index* for DL models on two GPUs (TITAN Xp and GTX 1080 Ti) using OpenZmeter and CodeCarbon. The values for the *Kappa Index*, energy consumption in kWh, and the *Kappa–Energy Index* are presented as averages and standard deviations, providing a detailed view of the efficiency of each energy meter. The results show that, similar to Section 3.1, CodeCarbon tends to underestimate energy consumption compared with OpenZmeter, resulting in a higher *Kappa–Energy Index*, especially on the GTX 1080 Ti. These differences are statistically significant ($p<3.07\times 10^{-6}$), highlighting variations in energy measurements between software and hardware energy meters in DL models.

The Kruskal–Wallis test, using OpenZmeter measurements (Table 8), compares the *Kappa–Energy Index* of the six architectures on both GPUs. For both GPUs, a ($p<1\times 10^{-7}$) is observed, indicating significant differences between architectures. The post hoc analysis with Dunn’s test confirms significant differences between specific pairs, such as AlexNet vs. VGG16, ResNet18 vs. VGG16, and Swin-T vs. ConvNeXt-T, with Bonferroni-adjusted *p*-values below 0.05 on both GPUs (Table 9). This analysis corroborates that AlexNet and ResNet18 are the most energy-efficient architectures across both GPUs, while ConvNeXt-T consistently ranks as the least efficient.

On the TITAN Xp, AlexNet demonstrates a 42.41% energy efficiency advantage over VGG16 and a marginal 1.24% gain over ResNet18, with no statistically significant difference between AlexNet and ResNet18. EfficientNet-B3 follows closely, showing competitive efficiency compared with the top models. Swin-T exhibits moderate efficiency, outperforming VGG16 but lagging behind AlexNet and ResNet18 by 23.56%. ConvNeXt-T is significantly less efficient, with a p<0.05 indicating substantial differences from all other models.

On the GTX 1080 Ti, ResNet18 emerges as the most efficient model, achieving a 41.60% energy efficiency improvement over VGG16 and a 4.06% advantage over EfficientNet-B3. AlexNet ranks second, trailing ResNet18 by only 3.50% but maintaining a significant lead over VGG16. Swin-T demonstrates moderate performance, outperforming ConvNeXt-T but remaining significantly less efficient than the top three architectures. ConvNeXt-T again ranks as the least efficient, with Dunn’s test confirming significant differences (p<0.05) from higher-performing models.

**Figure 13:**
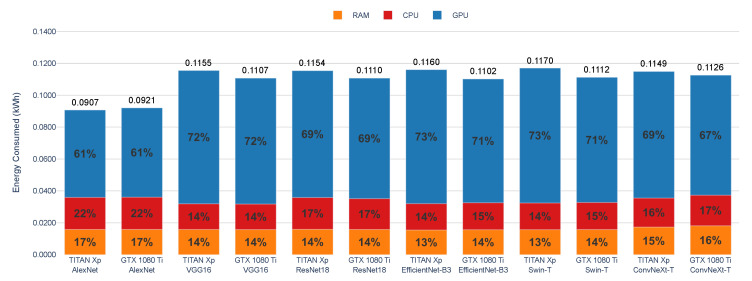
Energy consumption by component (RAM, CPU, GPU) during the training of AlexNet, VGG16, ResNet18, EfficientNet-B3, Swin-T, and ConvNeXt-T on TITAN Xp and GTX 1080 Ti GPUs. The results are obtained using CodeCarbon.

Figure 13 shows the energy consumption by component (RAM, CPU, and GPU) during the training of the DL models AlexNet, VGG16, ResNet18, EfficientNet-B3, Swin-T, and ConvNeXt-T on two different GPUs: TITAN Xp and GTX 1080 Ti. The stacked bars represent the percentage contribution of each component to the total energy consumption (in kWh) for each model and GPU combination. These results, obtained using CodeCarbon, indicate that the GPU accounts for the majority of energy consumption, contributing approximately 61–73%, depending on the DL model and GPU.

The CPU contributes 14–22%, while the RAM accounts for 13–17% of the total energy consumption across all models. The TITAN Xp shows slightly higher total energy consumption compared with the GTX 1080 Ti for most models, with the highest values recorded for Swin-T (0.1170 kWh) and EfficientNet-B3 (0.1160 kWh).

While ConvNeXt-T does not show the highest total energy consumption (0.1149 kWh on TITAN Xp and 0.1126 kWh on GTX 1080 Ti), its distribution highlights a larger contribution from CPU and RAM compared with other models. Swin-T exhibits the highest total energy consumption on the TITAN Xp, while ConvNeXt-T shows the highest consumption on the GTX 1080 Ti. EfficientNet-B3 follows closely in both cases. AlexNet has the lowest total energy consumption across both GPUs. Variations in energy consumption are primarily due to the computational demands of each model and the GPUs’ workload, which consistently dominates the energy profile.

#### 3.2.2. Evaluation of Kappa–Energy Index for DL Models Inference

The following analysis presents the inference process used to assess the energy efficiency of six selected DL models on two GPUs (TITAN Xp and GTX 1080 Ti). The dataset consists of 10 classes, each containing 100 images, with inference performed 15 times on the same GPU where each model is initially generated. Table 10 summarizes the results of the *Kappa–Energy Index* across different DL architectures. It includes execution time in seconds, the *Kappa Index* value, energy consumption in kWh using OpenZmeter, and the average *Kappa–Energy Index* with its corresponding standard deviation for each architecture and GPU combination.

Statistical analysis is conducted using the Kruskal–Wallis test to determine significant differences in KEI across architectures for both GPUs (p<0.0001). Pairwise comparisons with Dunn’s post hoc analysis reveal significant differences between several architectures. ResNet18 achieves the highest KEI on the TITAN Xp, while VGG16 and ConvNeXt-T record the lowest values. No significant difference is observed between Swin-T and VGG16. AlexNet and EfficientNet-B3 exhibit moderate KEI values, with no significant differences detected between them. On the GTX 1080 Ti, ResNet18 once again achieves the highest KEI, while VGG16 and ConvNeXt-T maintain the lowest values. Swin-T achieves better KEI than VGG16 and ConvNeXt-T but remains below AlexNet and EfficientNet-B3. These results confirm that ResNet18 demonstrates the highest energy efficiency during inference on both GPUs, whereas VGG16 and ConvNeXt-T show the least efficiency.

**Figure 14:**
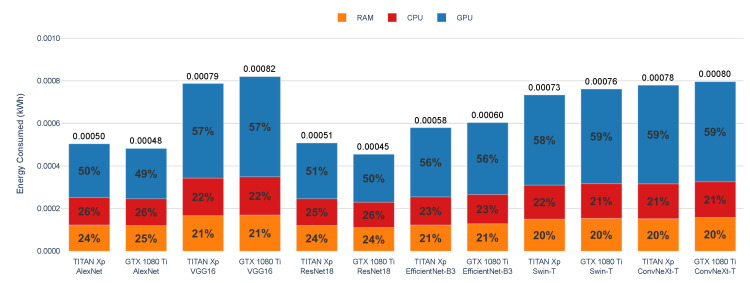
Energy consumption by component (RAM, CPU, GPU) during the inference of AlexNet, VGG16, ResNet18, EfficientNet-B3, Swin-T, and ConvNeXt-T on TITAN Xp and GTX 1080 Ti GPUs. The results are obtained using CodeCarbon.

Figure 14 shows the distribution of energy consumption across different hardware components (RAM, CPU, and GPU) during the inference of the six evaluated DL models on two GPU models: TITAN Xp and GTX 1080 Ti. The measurements, obtained using CodeCarbon, indicate each component’s contribution to the total energy consumed. As observed, the GPU consistently accounts for the largest share of energy usage, followed by the CPU and RAM.

The GPU contributes 49–59% of the total energy consumption during inference, slightly lower than in the training phase due to reduced computational intensity. The CPU accounts for 21–26%, while the RAM contributes 20–25%, consistently remaining the least demanding component. The TITAN Xp shows slightly higher energy consumption across all components compared with the GTX 1080 Ti, as observed in models like AlexNet, where the TITAN Xp’s GPU accounts for 50% of the total energy consumption versus 49% on the GTX 1080 Ti.

AlexNet exhibits the lowest total energy consumption during inference, consistent with its simpler architecture. VGG16 and EfficientNet-B3 follow, with higher energy demands and similar energy distribution patterns across components. ResNet18 demonstrates increased energy demand due to its greater complexity. Swin-T and ConvNeXt-T further highlight this trend, with GPUs contributing 58–59% of the total energy consumption across both GPUs. ConvNeXt-T shows the highest GPU contribution on the GTX 1080 Ti, while Swin-T exhibits the highest overall energy consumption on the TITAN Xp. CPU and RAM contributions remain consistent, with CPUs accounting for 21% and RAM for 20%.

### 3.3. Evaluation of Hyperparameter Influence and Energy Scaling per CUDA Core

The scalability and adaptability of the *Kappa–Energy Index* require an evaluation of its sensitivity to hyperparameters and hardware configurations. This section specifically examines the effect of batch size, a key hyperparameter, on energy consumption and model performance. To provide a hardware-agnostic perspective, energy consumption is normalized per CUDA core by dividing the NVML-measured energy consumption by the number of CUDA cores in each GPU. This normalization facilitates a detailed comparison of energy efficiency across architectures and hardware configurations.

Table 11 presents the results of the training phase, focusing on the *Kappa–Energy Index* and energy consumption metrics for different architectures and batch sizes on the TITAN Xp and GTX 1080 Ti GPUs. The analysis reveals that smaller batch sizes consistently yield higher energy efficiency compared with larger batch sizes across all architectures. For instance, on the TITAN Xp, AlexNet’s *Kappa–Energy Index* drops by approximately 14.2% when the batch size increases from 32 to 64. Similarly, VGG16, ResNet18, and EfficientNet-B3 exhibit reductions in energy efficiency of about 8.2%, 5.2%, and 6.2%, respectively, highlighting the additional energy cost of larger batch sizes. Swin-T follows a comparable trend, with a decrease of around 17.8%, while ConvNeXt-T shows a smaller decline of roughly 1.0%.

The results on the GTX 1080 Ti are consistent with those on the TITAN Xp, where larger batch sizes decrease energy efficiency for all architectures. While AlexNet and VGG16 experience the most significant reductions, ResNet18 shows better stability with a smaller decrease in its *Kappa–Energy Index*. Swin-T and ConvNeXt-T follow this pattern, with Swin-T showing a noticeable reduction, and ConvNeXt-T exhibiting a moderate decline as batch size increases.

The Energy Consumed per CUDA Core metric further highlights the hardware-specific effects of batch size and architecture on energy efficiency. On the TITAN Xp, AlexNet’s energy consumption per CUDA core decreases slightly with larger batch sizes, consistent with trends observed for other architectures, including VGG16 and ResNet18. Swin-T and ConvNeXt-T also exhibit small changes, with Swin-T showing a slight increase and ConvNeXt-T demonstrating minimal fluctuations. On the GTX 1080 Ti, energy consumption per CUDA core is generally higher, with VGG16 and EfficientNet-B3 showing the most notable increases. Swin-T and ConvNeXt-T follow a similar trend, with ConvNeXt-T showing a moderate increase, while Swin-T exhibits a slight decrease. ResNet18, however, demonstrates better scalability on both GPUs, maintaining relatively stable energy consumption per CUDA core as batch size grows.

## 4. Discussion

The experimental results obtained in our study reveal significant differences between hardware and software energy meters in DL models. The validation of energy measurement tools is one of the aspects addressed in this research, emphasizing the need for accurate calibration for reliable assessments. Hardware-based tools, such as OpenZmeter, provide high-precision component-level measurements and serve as benchmarks for validating software tools. Notably, software tools displayed up to a 10% variability in energy estimates, underscoring the importance of calibration to align with hardware standards.

The findings confirm the GPUs dominant role in energy consumption during both the training and inference phases. While AlexNet exhibits the lowest energy usage due to its simpler architecture, more complex models like VGG16 and EfficientNet-B3 demand significantly higher resources. This aligns with prior studies on the impact of model complexity on energy consumption [39]. However, the observed trade-offs between performance and energy usage highlight the critical role of hardware efficiency in optimizing Deep Learning workflows. Notably, the GTX 1080 Ti consistently demonstrates not only lower energy consumption but also higher Kappa and Kappa–Energy Index (KEI) values across most architectures when compared with the TITAN Xp. Swin-T and ConvNeXt-T further extend this analysis by illustrating the energy demands of emerging architectures, with ConvNeXt-T showing a slightly higher GPU dependency. In this regard, the growing complexity of balancing accuracy and energy efficiency in state-of-the-art models is illustrated by the findings.

The *Kappa–Energy Index* emerges as a pivotal metric for evaluating energy-performance trade-offs, providing a comprehensive view of model efficiency. ResNet18 consistently achieves the highest KEI scores across both GPUs, reflecting its ability to balance computational demands with accuracy. EfficientNet-B3 and AlexNet also demonstrate favorable KEI results, further validating the metric’s applicability. Statistical significance tests (p<0.001) confirm the robustness of these findings, offering a reliable framework for guiding architecture selection in energy-critical applications. The comparability of KEI values for Swin-T and ConvNeXt-T with EfficientNet-B3 suggests that emerging architectures are viable candidates for energy-sensitive deployments.

Finally, batch size analysis reveals a pronounced effect on energy efficiency, with smaller batch sizes consistently yielding higher efficiency across all architectures. ResNet18 and EfficientNet-B3 show statistically significant improvements in energy efficiency with smaller batches (p<0.01). These results emphasize the importance of hyperparameter optimization in reducing computational costs while maintaining performance. This finding aligns with previous research [32], highlighting batch size as a critical factor in deep learning optimization.

## 5. Conclusions

This study introduces the *Kappa–Energy Index* as a novel metric for evaluating the trade-off between energy efficiency and model performance in DL architectures. The results demonstrate the utility of this index across various scenarios, highlighting its adaptability to both training and inference phases. During training, AlexNet and ResNet18 exhibit superior KEI values, reflecting their balance between computational efficiency and accuracy, particularly when measured using OpenZmeter. EfficientNet-B3 follows closely, reinforcing its energy-efficient design, while VGG16 consistently shows lower efficiency, illustrating the impact of model complexity on energy demands.

In inference, the KEI values further emphasize the dominance of architectures like ResNet18 and EfficientNet-B3 in achieving optimal performance with reduced energy costs. The GTX 1080 Ti consistently outperforms the TITAN Xp in energy efficiency across all models, reaffirming the influence of hardware optimization on energy consumption. Swin-T and ConvNeXt-T, representing modern architectures, show competitive KEI values, indicating their potential for energy-sensitive deployments, albeit with increased complexity compared with classical architectures.

The validation of energy measurement tools reveals discrepancies between hardware-based and software-based meters. OpenZmeter serves as a reliable reference, while CodeCarbon and Carbontracker exhibit tendencies to under- or overestimate energy consumption. The results highlight the critical role of calibration and validation in order to ensure accurate energy assessments, particularly when comparing diverse architectures and hardware setups.

Future work should extend the application of the KEI metric to a broader range of DL models, including Recurrent Neural Networks and Generative Adversarial Networks, and explore its relevance in other domains such as natural language processing and time series analysis. Furthermore, the integration of KEI with real-time energy monitoring systems could facilitate dynamic adjustments in computational resource allocation, advancing the sustainability of AI workflows. In this way, our approach underscores the importance of standardized energy metrics in driving innovation and environmental responsibility within the field of machine learning.

## Figures and Tables

**Table 1:** Number of samples of each class.

Classes	Train	Test	Total
Cassette player	993	357	1350
Tench	963	387	1350
Garbage truck	961	389	1350
Parachute	960	390	1350
French horn	956	394	1350
English springer	955	395	1350
Golf ball	951	399	1350
Church	941	409	1350
Gas pump	931	419	1350
Chain saw	858	386	1244
**Total**	9469	3925	13,394

**Table 2:** Number of parameters and year of creation for the evaluated Neural Network Architectures.

Architecture	Number of Parameters	Year of Creation
AlexNet [52]	61,100,840	2012
VGG16 [53]	138,357,544	2014
ResNet18 [54]	11,689,512	2015
EfficientNet-B3 [55]	12,233,232	2019
Swin-T [9]	28,288,354	2021
ConvNeXt-T [56]	28,589,128	2022

**Table 3:** Cohen’s Kappa Agreement Levels.

Kappa Statistic	Agreement Levels
k<0	No agreement
0.01≤k≤0.20	Slight
0.21≤k≤0.40	Fair
0.41≤k≤0.60	Moderate
0.61≤k≤0.80	Substantial
0.81≤k≤1.00	Almost perfect

**Table 4:** Comparison of OpenZmeter, CodeCarbon, and Carbontracker.

Feature	OpenZmeter	CodeCarbon	Carbontracker
Type	Hardware and software	Software	Software
Energy Measurement	Reactive, active, apparent, RMS voltage/current, etc.	kWh during code execution	kWh during DL training
Carbon Emission Tracking	No	Yes (based on hardware and location)	Yes
Connectivity	Ethernet, Wi-Fi, 4G	API, Python libraries	Python libraries
Integration	Web interface, API	API, Python libraries	API, Python libraries
Focus	Energy and Power Quality Analysis	Carbon footprint reduction in software execution	Energy management in DL training
Advanced Features	Real-time alerts, power quality analysis	Reports, geographical data center insights	Dynamic adjustment of training epochs

**Table 5:** Summary of Hardware and Software Configuration.

Category	Specifications
**Hardware**	
Architecture	x86_64
Processors	2× Intel Xeon E5-2640 v4 @ 2.40 GHz, 10 cores each, 90 W TDP each
RAM	126 GB
GPUs	3× NVIDIA GTX 1080 Ti, 250 W TDP, 3584 CUDA cores, 11 GB each
	1× NVIDIA TITAN Xp, 250 W TDP, 3840 CUDA cores, 12 GB
Power Meters	OpenZmeter
**Software**	
Operating System	Ubuntu 24.04 LTS
Python Version	3.10.14
Pytorch Version	2.5.1
CodeCarbon Version	2.4.1
Carbontracker Version	1.2.5

**Table 6:** Training Setup Specifications.

Category	Specifications
Number of epochs	90
Batch size	64
Optimizer	Stochastic Gradient Descent (SGD)
Learning rate	0.001
Momentum	0.9
Weight decay	0.0001

**Table 7:** Energy consumption results and statistical validation using different hardware configurations.

GPU	Energy Meter	Architecture	Execution Times (s)		Energy Consumed (kWh)		*p*-Values
			Average	Std. Dev.	Average	Std. Dev.	
TITAN Xp	OpenZmeter (reference meter)	AlexNet	1198	4	0.1062	0.0005	-
		VGG16	1197	3	0.1319	0.0010	-
		ResNet18	1200	4	0.1332	0.0007	-
		EfficientNet-B3	1183	5	0.1314	0.0018	-
		Swin-T	1201	4	0.1318	0.0020	-
		ConvNeXt-T	1259	2	0.1273	0.0014	-
	CodeCarbon (evaluated meter)	AlexNet	1199	4	0.0907	0.0003	$p<2.96\times 10^{-6}$
		VGG16	1197	3	0.1155	0.0005	$p<2.94\times 10^{-6}$
		ResNet18	1201	4	0.1154	0.0005	$p<2.98\times 10^{-6}$
		EfficientNet-B3	1183	5	0.1160	0.0003	$p<2.64\times 10^{-6}$
		Swin-T	1201	5	0.1170	0.0002	$p<2.86\times 10^{-6}$
		ConvNeXt-T	1259	1	0.1149	0.0002	$p<2.95\times 10^{-6}$
	Carbontracker (evaluated meter)	AlexNet	1198	4	0.1218	0.0037	$p<3.04\times 10^{-6}$
		VGG16	1197	3	0.1537	0.0020	$p<2.98\times 10^{-6}$
		ResNet18	1201	4	0.1542	0.0025	$p<3.02\times 10^{-6}$
		EfficientNet-B3	1183	5	0.1545	0.0016	$p<3.01\times 10^{-6}$
		Swin-T	1201	5	0.1551	0.0019	$p<2.97\times 10^{-6}$
		ConvNeXt-T	1259	2	0.1509	0.0018	$p<3.05\times 10^{-6}$
GTX 1080 Ti	OpenZmeter (reference meter)	AlexNet	1200	3	0.1063	0.0011	-
		VGG16	1191	3	0.1225	0.0013	-
		ResNet18	1201	5	0.1247	0.0004	-
		EfficientNet-B3	1199	7	0.1210	0.0018	-
		Swin-T	1206	3	0.1238	0.0033	-
		ConvNeXt-T	1384	2	0.1247	0.0021	-
	CodeCarbon (evaluated meter)	AlexNet	1201	4	0.0921	0.0007	$p<3.04\times 10^{-6}$
		VGG16	1192	3	0.1107	0.0003	$p<4.51\times 10^{-6}$
		ResNet18	1201	5	0.1110	0.0003	$p<6.56\times 10^{-6}$
		EfficientNet-B3	1199	7	0.1102	0.0003	$p<2.95\times 10^{-6}$
		Swin-T	1206	3	0.1112	0.0001	$p<1.33\times 10^{-6}$
		ConvNeXt-T	1385	2	0.1126	0.0010	$p<3.00\times 10^{-6}$
	Carbontracker (evaluated meter)	AlexNet	1200	3	0.1241	0.0042	$p<3.04\times 10^{-6}$
		VGG16	1191	3	0.1454	0.0024	$p<3.04\times 10^{-6}$
		ResNet18	1201	5	0.1452	0.0042	$p<2.98\times 10^{-6}$
		EfficientNet-B3	1199	7	0.1450	0.0027	$p<3.02\times 10^{-6}$
		Swin-T	1206	3	0.1469	0.0020	$p<1.46\times 10^{-6}$
		ConvNeXt-T	1385	2	0.1492	0.0021	$p<3.04\times 10^{-6}$

Note: CodeCarbon and Carbontracker are the evaluated meters, while OpenZmeter is the reference energy meter.

**Table 8:** Training *Kappa–Energy Index* results and statistical validation.

GPU	Energy Meter	Architecture	Kappa		Energy Consumed (kWh)		*Kappa–Energy Index*		*p*-Values
			Average	Std. Dev.	Average	Std. Dev.	Average	Std. Dev.	
TITAN Xp	OpenZmeter	AlexNet	0.6427	0.0032	0.1062	0.0005	6.0518	0.0483	-
		VGG16	0.5602	0.0000	0.1319	0.0010	4.2472	0.0339	-
		ResNet18	0.7958	0.0023	0.1332	0.0007	5.9745	0.0281	-
		EfficientNet-B3	0.7256	0.0000	0.1314	0.0018	5.5221	0.0772	-
		Swin-T	0.5444	0.0000	0.1318	0.0020	4.1305	0.0601	-
		ConvNeXt-T	0.1567	0.0000	0.1273	0.0014	1.2310	0.0133	-
	CodeCarbon	AlexNet	0.6427	0.0032	0.0907	0.0003	7.0860	0.0528	$p<3.07\times 10^{-6}$
		VGG16	0.5602	0.0000	0.1155	0.0005	4.8502	0.0207	$p<3.07\times 10^{-6}$
		ResNet18	0.7958	0.0023	0.1154	0.0005	6.8960	0.0273	$p<3.07\times 10^{-6}$
		EfficientNet-B3	0.7256	0.0000	0.1160	0.0003	6.2552	0.0149	$p<3.07\times 10^{-6}$
		Swin-T	0.5444	0.0000	0.1170	0.0002	4.6530	0.0103	$p<3.07\times 10^{-6}$
		ConvNeXt-T	0.1567	0.0000	0.1149	0.0002	1.3638	0.0031	$p<3.07\times 10^{-6}$
GTX 1080 Ti	OpenZmeter	AlexNet	0.6545	0.0012	0.1063	0.0011	6.1571	0.0694	-
		VGG16	0.5515	0.0000	0.1225	0.0013	4.5020	0.0479	-
		ResNet18	0.7946	0.0031	0.1247	0.0004	6.3721	0.0361	-
		EfficientNet-B3	0.7411	0.0000	0.1210	0.0018	6.1248	0.0682	-
		Swin-T	0.4867	0.0000	0.1238	0.0033	3.9313	0.1005	-
		ConvNeXt-T	0.1600	0.0000	0.1247	0.0021	1.2831	0.0219	-
	CodeCarbon	AlexNet	0.6545	0.0012	0.0921	0.0007	7.1064	0.0595	$p<3.07\times 10^{-6}$
		VGG16	0.5515	0.0000	0.1107	0.0003	4.9819	0.0156	$p<3.07\times 10^{-6}$
		ResNet18	0.7937	0.0024	0.1110	0.0003	7.1505	0.0363	$p<3.07\times 10^{-6}$
		EfficientNet-B3	0.7411	0.0000	0.1102	0.0003	6.7250	0.0156	$p<3.07\times 10^{-6}$
		Swin-T	0.4867	0.0000	0.1112	0.0001	4.3768	0.0042	$p<3.07\times 10^{-6}$
		ConvNeXt-T	0.1600	0.0000	0.1126	0.0010	1.4210	0.0219	$p<3.07\times 10^{-6}$

**Table 9:** Post Hoc Analysis: Dunn’s Test Results for DL Models on Different GPU Models.

GPU	Comparison	AlexNet	VGG16	ResNet18	EfficientNet-B3	Swin-T	ConvNeXt-T
TITAN Xp	AlexNet	-	$5.85\times 10^{-5}$	-	$3.50\times 10^{-2}$	$9.07\times 10^{-9}$	$1.26\times 10^{-13}$
	VGG16	$5.85\times 10^{-5}$	-	$1.75\times 10^{-2}$	-	-	$2.49\times 10^{-2}$
	ResNet18	-	$1.75\times 10^{-2}$	-	-	$2.16\times 10^{-5}$	$2.46\times 10^{-9}$
	EfficientNet-B3	$3.50\times 10^{-2}$	-	-	-	$2.49\times 10^{-2}$	$3.57\times 10^{-5}$
	Swin-T	$9.07\times 10^{-9}$	-	$2.16\times 10^{-5}$	$2.49\times 10^{-2}$	-	-
	ConvNeXt-T	$1.26\times 10^{-13}$	$2.49\times 10^{-2}$	$2.46\times 10^{-9}$	$3.57\times 10^{-5}$	-	-
GTX 1080 Ti	AlexNet	-	$1.34\times 10^{-1}$	$4.08\times 10^{-1}$	-	$1.09\times 10^{-3}$	$2.91\times 10^{-7}$
	VGG16	$1.34\times 10^{-1}$	-	$2.11\times 10^{-5}$	$4.08\times 10^{-1}$	-	$4.03\times 10^{-2}$
	ResNet18	$4.08\times 10^{-1}$	$2.11\times 10^{-5}$	-	$1.34\times 10^{-1}$	$1.64\times 10^{-8}$	$7.57\times 10^{-14}$
	EfficientNet-B3	-	$4.08\times 10^{-1}$	$1.34\times 10^{-1}$	-	$5.27\times 10^{-3}$	$2.83\times 10^{-6}$
	Swin-T	$1.09\times 10^{-3}$	-	$1.64\times 10^{-8}$	$5.27\times 10^{-3}$	-	-
	ConvNeXt-T	$2.91\times 10^{-7}$	$4.03\times 10^{-2}$	$7.57\times 10^{-14}$	$2.83\times 10^{-6}$	-	-

Note: Bonferroni-adjusted *p*-values (p<0.05) for both GPUs.

**Table 10:** Inference *Kappa–Energy Index* results and statistical validation (OpenZmeter).

GPU	Architecture	Execution Times (s)	Kappa	Energy Consumed (kWh)	*Kappa–Energy Index*	
					Average	Std. Dev.
TITAN Xp	AlexNet	8.6000	0.6600	0.00060	1100.0000	117.4749
	VGG16	11.8000	0.5589	0.00090	621.0000	53.3947
	ResNet18	8.4667	0.8089	0.00060	1348.1667	152.5554
	EfficientNet-B3	8.6667	0.7256	0.00070	1036.5714	87.6063
	Swin-T	10.6000	0.5444	0.00080	680.5000	50.2012
	ConvNeXt-T	10.7333	0.1567	0.00090	174.1111	14.2351
GTX 1080 Ti	AlexNet	8.0000	0.6744	0.00060	1124.0000	102.8994
	VGG16	12.0667	0.5633	0.00100	563.3000	32.3208
	ResNet18	7.8000	0.8156	0.00050	1631.2000	140.3914
	EfficientNet-B3	9.1333	0.7411	0.00070	1058.7143	90.5074
	Swin-T	10.8000	0.4867	0.00090	540.7778	30.9418
	ConvNeXt-T	11.2000	0.1600	0.00090	177.7778	9.4729

**Table 11:** Hyperparameter Influence on Energy Efficiency and CUDA Core Energy Scaling Analysis.

GPU	Architecture	Batch Size	Kappa	Energy Consumed	Kappa-Energy	Energy Consumed	Energy-Cuda
			Average	(Global, kWh) ^*^	Index (Global)	(NVML, kWh) ^†^	Core (NVML) ^†^
TITAN Xp	AlexNet	32	0.7756	0.1100	7.0509	0.0600	$1.5625\times 10^{-5}$
		64	0.6427	0.1062	6.0518	0.0548	$1.4271\times 10^{-5}$
	VGG16	32	0.6244	0.1349	4.6286	0.0862	$2.2448\times 10^{-5}$
		64	0.5602	0.1319	4.2472	0.0836	$2.1771\times 10^{-5}$
	ResNet18	32	0.8417	0.1335	6.3049	0.0818	$2.1302\times 10^{-5}$
		64	0.7958	0.1332	5.9745	0.0795	$2.0703\times 10^{-5}$
	EfficientNet-B3	32	0.7763	0.1319	5.8855	0.0851	$2.2161\times 10^{-5}$
		64	0.7256	0.1314	5.5221	0.0840	$2.1875\times 10^{-5}$
	Swin-T	32	0.6411	0.1276	5.0243	0.0820	$2.1354\times 10^{-5}$
		64	0.5444	0.1318	4.1305	0.0846	$2.2031\times 10^{-5}$
	ConvNeXt-T	32	0.1633	0.1314	1.2428	0.0825	$2.1484\times 10^{-5}$
		64	0.1567	0.1273	1.2310	0.0793	$2.0651\times 10^{-5}$
GTX 1080 Ti	AlexNet	32	0.7867	0.1064	7.3938	0.0580	$1.6183\times 10^{-5}$
		64	0.6545	0.1063	6.1571	0.0560	$1.5625\times 10^{-5}$
	VGG16	32	0.6411	0.1242	5.1618	0.0813	$2.2684\times 10^{-5}$
		64	0.5515	0.1225	4.5020	0.0789	$2.2015\times 10^{-5}$
	ResNet18	32	0.8375	0.1172	7.1459	0.0706	$1.9699\times 10^{-5}$
		64	0.7946	0.1247	6.3721	0.0755	$2.1066\times 10^{-5}$
	EfficientNet-B3	32	0.7444	0.1180	6.3085	0.0756	$2.1094\times 10^{-5}$
		64	0.7411	0.1210	6.1248	0.0776	$2.1652\times 10^{-5}$
	Swin-T	32	0.5882	0.1237	4.7551	0.0801	$2.2349\times 10^{-5}$
		64	0.4867	0.1238	3.9313	0.0784	$2.1875\times 10^{-5}$
	ConvNeXt-T	32	0.1744	0.1168	1.4932	0.0711	$1.9838\times 10^{-5}$
		64	0.1600	0.1247	1.2831	0.0752	$2.0982\times 10^{-5}$

^*^ Measurement includes the entire system (CPU, GPU, RAM, etc.). ^†^ Measurement specific to the GPU using NVML.

## Data Availability

Data are contained within the article. Additional details and datasets can be found at the following GitHub repository: https://github.com/seriab/Deep-Learning-Energy-Consumption-Index.
